# Antibody-dependent cellular cytotoxicity response to SARS-CoV-2 in COVID-19 patients

**DOI:** 10.1038/s41392-021-00759-1

**Published:** 2021-09-24

**Authors:** Yuanling Yu, Meiyu Wang, Xiaoai Zhang, Shufen Li, Qingbin Lu, Haolong Zeng, Hongyan Hou, Hao Li, Mengyi Zhang, Fei Jiang, Jiajing Wu, Ruxia Ding, Zehua Zhou, Min Liu, Weixue Si, Tao Zhu, Hangwen Li, Jie Ma, Yuanyuan Gu, Guangbiao She, Xiaokun Li, Yulan Zhang, Ke Peng, Weijin Huang, Wei Liu, Youchun Wang

**Affiliations:** 1 Division of HIV/AIDS and Sex-transmitted Virus Vaccines, Institute for Biological Product Control, National Institutes for Food and Drug Control (NIFDC), Beijing, China; 2 Graduate School of Peking Union Medical College, Chinese Academy of Medical Sciences, Peking Union Medical College, Beijing, China; 3 State Key Laboratory of Pathogen and Biosecurity, Beijing Institute of Microbiology and Epidemiology, Beijing, China; 4 State Key Laboratory of Virology, Wuhan Institute of Virology, Chinese Academy of Sciences, Wuhan, Hubei China; 5 Department of Laboratorial Science and Technology, School of Public Health, Peking University, Beijing, China; 6 Department of Laboratory Medicine, Tongji Hospital, Tongji Medical College, Huazhong University of Science and Technology, Wuhan, China; 7 Sinovac Biotech Co., Ltd, Beijing, China; 8 Cansino Biotech Incorporation, Tianjin, China; 9 Stemirna Therapeutics, Ltd, Shanghai, China; 10 Anhui Zhifeilongcom Biopharmaceutical Co., Ltd, Hefei, China; 11 University of Chinese Academy of Sciences, Beijing, China

**Keywords:** Vaccines, Infectious diseases

## Abstract

Antibody-dependent cellular cytotoxicity (ADCC) responses to viral infection are a form of antibody regulated immune responses mediated through the Fc fragment. Whether severe acute respiratory syndrome coronavirus 2 (SARS-CoV-2) triggered ADCC responses contributes to COVID-19 disease development is currently not well understood. To understand the potential correlation between ADCC responses and COVID-19 disease development, we analyzed the ADCC activity and neutralizing antibody response in 255 individuals ranging from asymptomatic to fatal infections over 1 year post disease. ADCC was elicited by 10 days post-infection, peaked by 11–20 days, and remained detectable until 400 days post-infection. In general, patients with severe disease had higher ADCC activities. Notably, patients who had severe disease and recovered had higher ADCC activities than patients who had severe disease and deceased. Importantly, ADCC activities were mediated by a diversity of epitopes in SARS-COV-2-infected mice and induced to comparable levels against SARS-CoV-2 variants of concern (VOCs) (B.1.1.7, B.1.351, and P.1) as that against the D614G mutant in human patients and vaccinated mice. Our study indicates anti-SARS-CoV-2 ADCC as a major trait of COVID-19 patients with various conditions, which can be applied to estimate the extra-neutralization level against COVID-19, especially lethal COVID-19.

## Introduction

Since its emergence in 2019, severe acute respiratory syndrome coronavirus 2 (SARS-CoV-2), which is the etiology of coronavirus disease 2019 (COVID-19), has rapidly spread and caused >169 million cases and 3.5 million deaths globally by 1 June 2021.^1^ Recently, the progress in controlling the pandemic is being dampened by the emergence of variants that are more transmissible and escape control by both vaccine-induced and convalescent immune protection.^2,3^ The challenge remains to identify immune correlates of protection against SARS-CoV-2 infection or vaccination. However, the current knowledge of the mechanisms by which antibodies can protect against COVID-19 infection and disease or, conversely, contribute to disease development, remain obscure. This knowledge gap has hampered the rational design of effective therapeutic interventions, as well as safe and effective vaccines against COVID-19.

While neutralizing antibodies (Nabs) can interfere with viral infection, non-Nabs mediated by Fc receptor binding to immune cells, can contribute to antiviral activities with mechanisms including antibody-medicated complement-dependent cytotoxicity, antibody-dependent cellular cytotoxicity (ADCC), and antibody-dependent cellular phagocytosis.^4^ Natural killer (NK) cells, neutrophils, monocytes, and macrophages are important innate effector cells of inducing ADCC effect in vitro. The major contributors to ADCC in vivo have been proposed to be NK cells, with the induction of the release of cytotoxic granules resulting in killing of infected cells.^5^ The importance of Fc-mediated ADCC functions in both protection and pathogenesis have been recently reported for various infectious pathogens.^6–9^ ADCC-inducing human immunodeficiency virus (HIV)-specific antibodies were identified as a key correlate of protection in the RV144 HIV vaccine trial.^10^ ADCC by engaging the Fc effector on NK cells or phagocytes also contributes to prevention against several viral infections including Dengue fever and Ebola.^11,12^ However, in terms of influenza, there has been much debate about the role of ADCC, with some studies that indicate a protective capacity of ADCC-inducing antibodies,^13^ whereas others have shown no role or exaggeration of the immune response by ADCC.^14–16^

In the context of COVID-19, functional non-Nab responses to SARS-CoV-2, which include ADCC, have been preliminarily investigated mostly by cross-sectional analyses. Tso et al. analyzed plasma from 3 uninfected controls and 20 subjects exposed to or recovering from SARS-CoV-2 infection and found that both neutralizing and non-neutralizing COVID-19 plasmas mediate ADCC.^17^ Another study that examined 95 SARS-COV-2-infected subjects revealed elevated ADCC activities in COVID-19 patients, especially hospitalized patients.^1^ Chen et al. performed a short-term longitudinal study of up to 1–2 months post infection, which revealed that 56 of the 61 convalescent serum samples induced ADCC-dependent elimination of target cells expressing SARS-CoV-2 spike (S), whereas none of the 15 healthy controls had detectable ADCC.^18^ Wen et al. extended the observation up to 149 days post-symptom onset, which revealed detectable ADCC activity, with S-specific antibodies that mediated more potent ADCC even when Nabs were decaying.^19^ In a cohort of SARS-CoV-2-recovered individuals, Anand et al. observed that ADCC activity decreased gradually between 6 weeks and 8 months post-symptom onset.^20^ However, the evolution of such activities during long convalescence from COVID-19 or whether ADCC functions track with differential disease severity is currently unknown.

In the current study, we analyzed serial serum samples that were prospectively collected from a cohort of patients and profiled the longitudinal pattern of ADCC during hospitalization and after convalescence of the disease. To examine the effect of ADCC against emerging SARS-CoV-2 mutants, we also explored the differential and qualitative features of variants of concern (VOCs) in eliciting ADCC by testing samples from patients and SARS-CoV-2-immunized mice.

## Results

### SARS-CoV-2-specific ADCC following infection

**Fig. 1:**
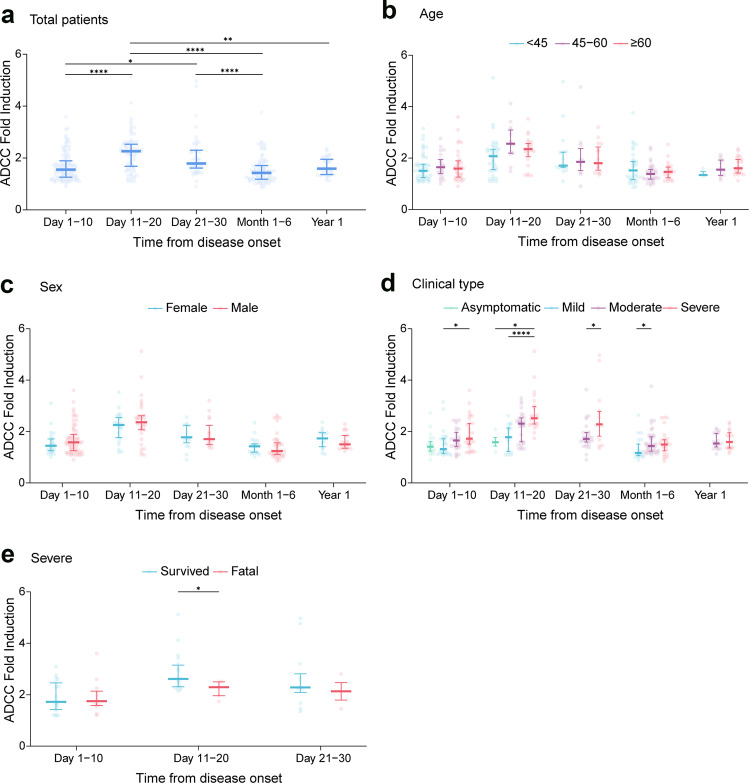
Antibody-dependent cellular cytotoxicity (ADCC) in COVID-19 patients over time. **a** Total patients (*n* = 255). **b** Patients stratified by age groups (97 patients were <40 years old; 74 patients were 40–60 years old; 84 patients were ≥60 years old). **c** Patients stratified by sex (164 males and 91 females). **d** Patients stratified by clinical phenotype (21 asymptomatic, 58 mild, 94 moderate, and 82 severe). **e** Patients stratified by clinical outcomes (30 survived and 19 fatal). Dots represent values for individual detection, and lines and error bars indicate the median and interquartile range, respectively. In **a**, **c**, **e**, Mann–Whitney *U* test was performed. In **b**, **d**, the multiple comparisons among the groups were made using Kruskal–Wallis test followed by Bonferroni post hoc correction; *P < 0.05, **P < 0.01, ****P < 0.0001. COVID-19 coronavirus disease 2019

A total of 341 serum samples from 234 patients (82 had severe disease, 94 had moderate disease, and 58 had mild disease) and 21 asymptomatic individuals with SARS-CoV-2 infection were tested for ADCC (Table 1). All 255 individuals, regardless of clinical phenotype, had detectable ADCC activity, that ranged from 0.85- to 5.12-fold induction. When all sampling points of the patients were combined for analysis, ADCC activity reached its peak at days 11–20, decreased thereafter, and maintained with comparable level at the last observation of 1 year post disease (Fig. 1a and Table 2). Moreover, we observed a similarly dynamic pattern across three age groups (Fig. 1b), and between males and females (Fig. 1c), i.e., all peaking at days 11–20, decreasing thereafter, and maintained with comparable level at the last observation.

**Table 1:** Basic characteristics of the COVID-19 patients

Variable	All patients (*N* = 255)	Survived (*n* = 236)	Fatal (*n* = 19)	*P* value
Age, years, median (IQR)	49 ± 16	49 ± 16	58 ± 14	0.007^a^
Sex, *n* (%)				0.060^b^
Male	164 (64.3)	148 (62.7)	16 (84.2)	
Female	91 (35.7)	88 (37.3)	3 (15.8)	
Clinical type, *n* (%)				<0.001^b^
Asymptomatic	21 (8.2)	21 (8.9)	0 (0)	
Mild	58 (22.8)	58 (24.6)	0 (0)	
Moderate	94 (36.9)	94 (39.8)	0 (0)	
Severe	82 (32.1)	63 (26.7)	19 (100)	

^a^The comparison was performed by Kruskal–Wallis rank test

^b^The comparison was performed by Fisher exact test

**Table 2:** The factors related to the levels of ADCC fold induction and neutralization ED_50_ among the COVID-19 patients by generalized estimation equation

Factors	ADCC fold induction		Neutralization ED_50_^a^	
	*β* (95% CI)	*P*	*β* (95% CI)	*P*
Age, years				
<45	1.00 (reference)		1.00 (reference)	
45–60	0.042 (−0.112, 0.197)	0.591	0.060 (−0.096, 0.216)	0.452
≥60	−0.053 (−0.208, 0.101)	0.499	0.119 (−0.045, 0.282)	0.155
Sex				
Male	1.00 (reference)		1.00 (reference)	
Female	−0.079 (−0.218, 0.06)	0.267	−0.016 (−0.154, 0.123)	0.823
Clinical type				
Asymptomatic	1.00 (reference)		1.00 (reference)	
Mild	0.153 (−0.124, 0.43)	0.278	−0.244 (−0.508, 0.020)	0.070
Moderate	0.265 (0.001, 0.529)	0.049	0.094 (−0.159, 0.347)	0.468
Severe	0.549 (0.279, 0.818)	<0.001	0.225 (−0.036, 0.486)	0.091
Day from symptom onset to sampling				
1−10 days	1.00 (reference)		1.00 (reference)	
11–20 days	0.459 (0.299, 0.619)	<0.001	0.142 (−0.006, 0.290)	0.060
21–30 days	0.226 (0.021, 0.432)	0.031	0.393 (0.253, 0.532)	<0.001
1–6 months	−0.292 (−0.445, −0.138)	<0.001	0.234 (0.081, 0.388)	0.003
1 year	−0.047 (−0.259, 0.165)	0.665	−0.035 (−0.254, 0.185)	0.756

*ADCC* antibody-dependent cell-mediated cytotoxicity, *CI* confidence interval, *COVID-19* coronavirus disease 2019

^a^The value of neutralization ED_50_ was log_10_-transformed

Differences in ADCC activity toward SARS-CoV-2 in relation to sex, age, and clinical phenotype were evaluated, which revealed no significant differences between males and females or across the three age groups at each time point (all *P* > 0.05; Fig. 1b, c). However, we indeed observed a clear trend of increased ADCC with more severe disease (Fig. 1d). For the peaking point at days 11–20, the severe cases displayed the highest ADCC (median: 2.52; interquartile range (IQR): 2.29–2.97), which was reduced to 2.31 (IQR: 1.60–2.53) in the moderate infection group and further reduced to 1.78 (IQR: 1.22–2.14) in the mild infection and 1.59 (IQR: 1.43–1.77) in the asymptomatic group. This trend in relation to disease severity was likewise observed across the whole observation, however, with the gratitude reduced as disease progressed. For example, significant difference between severe and mild group was observed at the first two time points, i.e., days 1–10, days 11–20 post disease, but turned insignificant at 1–6 months. Multivariate logistic regression analysis further confirmed the association between disease severity and ADCC, with significance attained for moderate disease (*P* = 0.049) and severe disease (*P* < 0.001) in comparison with asymptomatic infection (Table 2).

Among the 255 individuals, 82 had developed severe disease, which included 19 fatal cases who had developed severe disease during hospitalization. Compared with surviving patients, a significantly older age was found in fatal patients (Kruskal–Wallis rank test, *P* = 0.007; Table 1). We further performed subgroup analysis by comparing the production of ADCC from cases who had severe disease but developed different outcome. It is shown that severe patients who had survived displayed significantly higher ADCC at days 11–20 than those with fatal outcome (*P* = 0.044; Fig. 1e). The pattern of higher ADCC in surviving cases had remained at days 21–30, although without significance.

**Fig. 2:**
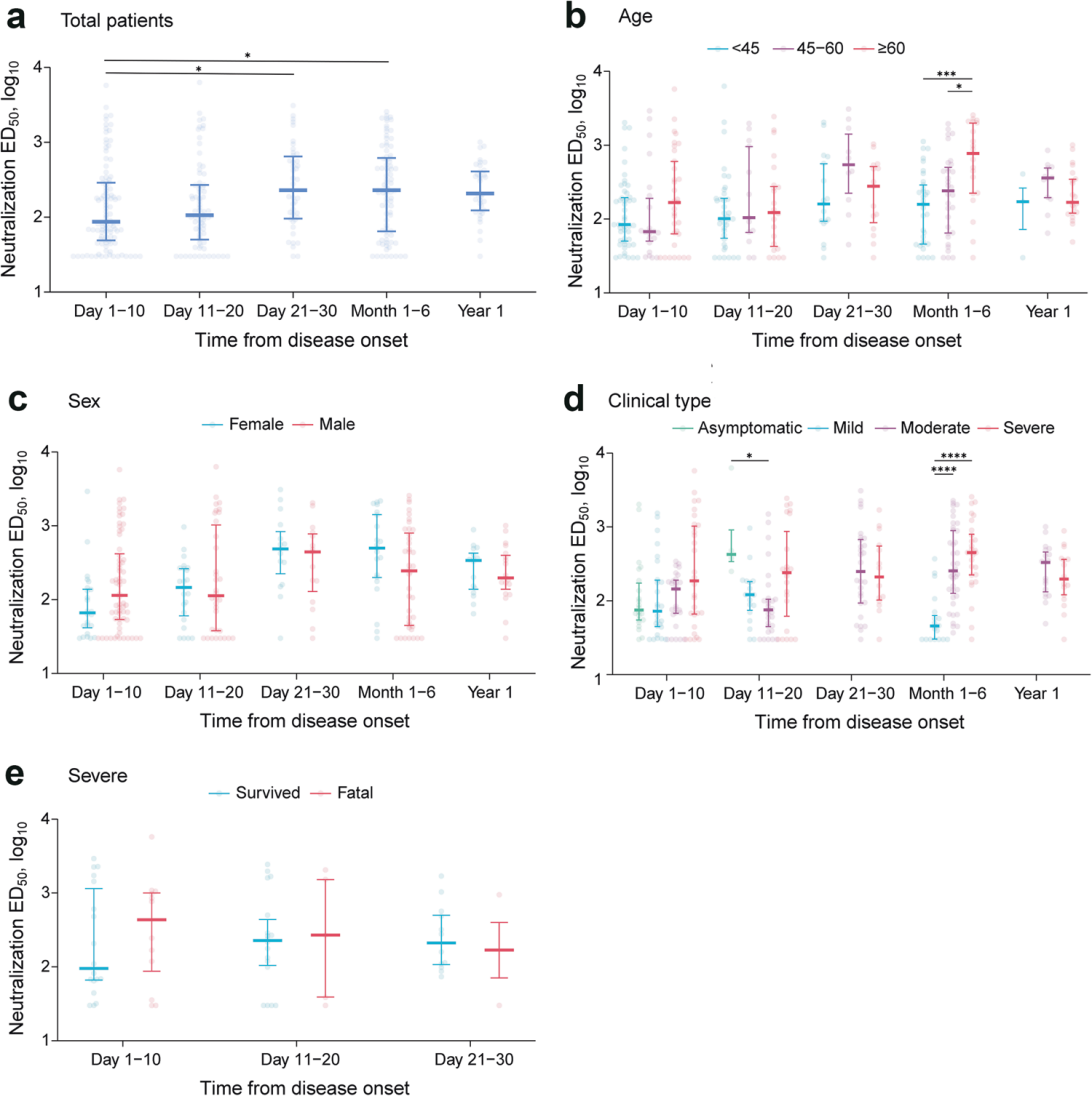
Neutralization antibodies in COVID-19 patients over time. **a** Total patients (*n* = 255). **b** Patients stratified by age groups (97 patients were <40 years old; 74 patients were 40–60 years old; 84 patients were ≥60 years old). **c** Patients stratified by sex (164 males and 91 females). **d** Patients stratified by clinical phenotype (21 asymptomatic, 58 mild, 94 moderate, and 82 severe). **e** Patients stratified by clinical outcomes (30 survived and 19 fatal). Dots represent values (log_10_-transformed) for individual detection, and lines and error bars indicate the median and interquartile range, respectively. In **a**, **c**, **e**, Mann–Whitney *U* test was performed. In **b**, **d**, the multiple comparisons among the groups were made using Kruskal–Wallis test followed by Bonferroni post hoc correction; *P < 0.05, ***P < 0.001, ****P < 0.0001. COVID-19 coronavirus disease 2019

The dynamics of Nab production after SARS-CoV-2 infection were similarly profiled. For all evaluated patients and asymptomatic individuals, 88.68% (227/255) had detectable Nabs. When all points from patients were combined, the Nabs reached their peak at days 21–30, decreased slightly thereafter, and were maintained with comparable level till the last time point sampled at 1 year post disease (Fig. 2a). Multivariate logistic regression analysis further confirmed that the highest Nab level was attained at days 21–30 (*P* < 0.001; Table 2). The level of Nab in relation to sex, age, and clinical phenotype was evaluated, which revealed no consistent trend in the differences between males and females or across the three age groups at each time point (Fig. 2b–d). Notably, higher Nabs in the severe and moderate groups than in the mild group was only significant at 1–6 months (*P* < 0.001; Fig. 2d). Subgroup analysis was performed by comparing the production of Nabs in severe cases who had fatal vs. non-fatal outcomes, which revealed comparable Nab level at each time point (Fig. 2e).

### Long-term follow-up of ADCC activities in a case cohort

The long-term profile of ADCC activities was further delineated in a case cohort of 44 patients who contributed ≥2 samples (Supplementary Fig. S1). On the basis of the sampling points, the specimens were grouped into two stages (within 30 days vs. >30 days post-symptom onset or turning point for positivity). In the first stage, 46 specimens from 16 patients (6 severe, 8 moderate, and 2 mild patients) and 2 asymptomatic individuals were tested. The median ADCC evaluated during 1–7 days was 1.21 (IQR: 1.19–1.94), which was significantly elevated to the highest level of 2.50 (IQR: 1.75–2.64) at 8–14 days (*P* = 0.024) and declined thereafter to 2.34 (IQR: 2.06–2.64) at 15–21 days, a comparable level to that evaluated during the later convalescent phase of 22–30 days (median, 2.21 IQR: 1.63–2.25; *P* = 0.826; Supplementary Fig. S1a). Thus, the trend during the first month of disease resembled that of the cross-sectional samples, which further verified the dynamic pattern of ADCC activity at late acute and early convalescent phases.

In the second stage, 56 paired samples obtained >1 month apart were collected from 28 patients (12 severe and 16 moderate) between 31 and 374 days. Either an increase or decrease of ADCC activity was observed in the same patient, regardless of their disease severity (Supplementary Fig. S1b). It was notable that a dramatic decrease in ADCC from 4 months to 374 days post disease was observed in 1 severe patient.

### ADCC responses elicited by the SARS-CoV-2 receptor binding domain (RBD) and S2 subunit

**Fig. 3:**
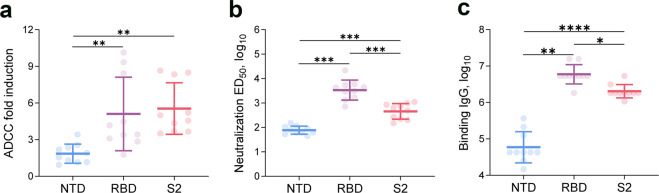
Antibody and ADCC activities mediated by different domains of SARS-COV-2 spike (S) protein in mice. The SARS-COV-2 N-terminal domain (NTD), receptor-binding domain (RBD), and S2 subunit were analyzed. **a** ADCC fold induction in the three domains. **b** Neutralization ED50 (log10-transformed) in the three domains. **c** Binding IgG (log10-transformed) in the three domains. The mean and standard deviation are shown. Comparisons among the three groups were performed by one-way analysis of variance; *P < 0.05, **P < 0.01, ***P < 0.001, ****P < 0.0001. ADCC antibody-dependent cell-mediated cytotoxicity, SARS-COV-2 severe acute respiratory syndrome coronavirus 2

The main target of COVID-19 vaccine is the S protein, that plays a vital role in virus attachment and entry into host cells. The S protein is composed of two subunits, S1 and S2. Although the RBD of S1 is immunodominant, as N-terminal domain (NTD)-specific and S2-specific neutralizing monoclonal antibodies (mAbs) are characterized, the antigenicity of NTD and S2 subunit have been described. To evaluate the role of the NTD, RBD, and S2 in activating ADCC, we assessed binding immunoglobulin G (IgG) antibodies, Nabs, and ADCC activity in the sera from NTD-, RBD-, or S2 protein-immunized mice. Compared with NTD, the RBD and S2 subunit mediated a higher ADCC induction fold (*P* = 0.007 for RBD vs. NTD; *P* = 0.002 for S2 vs. NTD; Fig. 3a). The S2 subunit mediated an even higher ADCC induction fold than RBD, but without significance (*P* = 0.894, Fig. 3a). Likewise, the Nabs against both RBD and S2 subunit were higher than those against the NTD (both *P* < 0.001), while the S2 subunit elicited lower Nabs than RBD (*P* < 0.001, Fig. 3b). Highly similar results were obtained for the binding IgG antibody to that of Nabs (i.e., all significant higher level for RBD vs. NTD; S2 vs. NTD, RBD vs. S2; Fig. 3c).

### ADCC activity of patients and immunized mice against VOCs

**Fig. 4:**
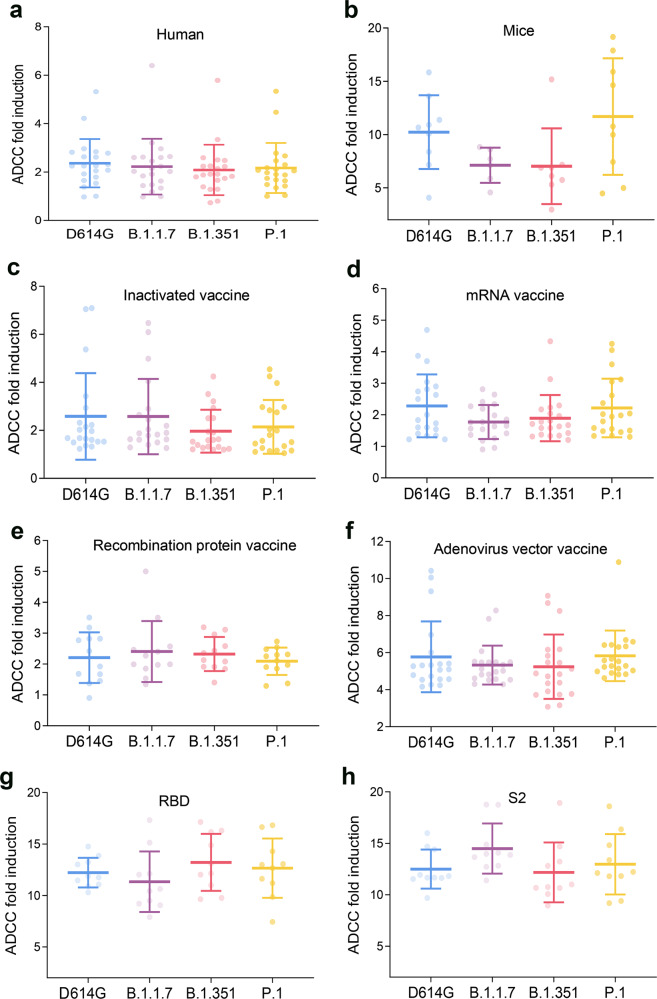
ADCC activity in human patients and mice infected with SARS-COV-2 variants or inoculated with various vaccines. Four SARS-COV-2 strains, which included D614G, B.1.1.7, B.1.351, and P.1, were used. Four vaccines, which included inactivated vaccine, recombination protein vaccine, mRNA vaccine, and adenovirus vector vaccine, were used. **a** ADCC activity in patients against D614G, B.1.1.7, B.1.351, and P.1. **b** ADCC activity in BALB/c mice infected with SARS-COV-2 pseudoviruses D614G, B.1.1.7, B.1.351, and P.1. **c** ADCC activity in the BALB/c mice inoculated with inactivated vaccine. **d** ADCC activity in BALB/c mice inoculated with the mRNA vaccine. **e** ADCC activity in BALB/c mice inoculated with the recombination protein vaccine. **f** ADCC fold induction in BALB/c mice inoculated with the adenovirus vector vaccine. **g** ADCC fold induction in BALB/c mice immunized with the SARS-COV-2 RBD proteins. **h** ADCC fold induction in the BALB/c mice immunized with SARS-COV-2 S2 protein. The mean and standard deviation are shown. Comparisons among the three groups were performed by one-way analysis of variance. ADCC antibody-dependent cell-mediated cytotoxicity, SARS-COV-2 severe acute respiratory syndrome coronavirus 2

To compare differences among the D614G reference strain and three SARS-CoV-2 VOCs (B.1.1.7 variant, B.1.351 variant, and P.1 variant) in term of their ability to trigger the ADCC response, we tested sera collected from patients and mice inoculated with SARS-CoV-2 pseudotyped viruses. Expression of S protein of SARS-CoV-2 variants on the surface of target cells was measured by flow cytometry. The percentages of S protein-positive cells were comparable, which ranged from 46.0 to 58.6% among target cells (Supplementary Fig. S2). In 21 randomly selected sera (17 males, median ages 47 years, 7 severe, collected at 1–151 days post disease), highly comparable ADCC activities were observed when 3 VOCs were applied in the tests (Fig. 4a). In contrast, slightly higher ADCC activities were measured in P.1-immunized mice than the B.1.1.7 variant- and B.1.351 variant-immunized mice and with a comparable level with those in D614G-immunized mice, however, with the difference attaining no significance (P.1 vs. B.1.1.7, *P* = 0.149; P.1 vs. B.1.351, *P* = 0.095, P.1 vs. D614G, *P* = 0.861; Fig. 4b). Compared with D614G reference strain immunized mice, a comparable ADCC response was found in B.1.1.7 variant and B.1.351 variant-immunized mice (B.1.1.7 vs. D614G, *P* = 0.454; B.1.351 vs. D614G, *P* = 0.359; Fig. 4b).

We next assessed serum from mice vaccinated with inactivated vaccine, adenovirus vector vaccine, mRNA vaccine, and recombinant protein subunit vaccine in terms of their difference in ADCC activity against VOCs (B.1.1.7, B.1.351, and P.1 variants) compared with the D614G reference strain. Similar ADCC activity was elicited against the three SARS-CoV-2 variants, all comparable with that of the D614G strain, which was observed in mice that received any type of the vaccination (Fig. 4c–f). When compared among vaccine types, significant higher ADCC activities were observed in mice immunized by the adenovirus vector vaccine than by the other three vaccines. This trend was consistently observed for all four evaluated strains (all *P* > 0.05; Fig. 4c–f).

SARS-CoV-2 RBD protein-immunized mouse sera were collected from mice immunized with 20 µg RBD proteins. The immunization dose of antigen was higher than that in mice vaccinated with the recombinant protein vaccine, which was 2 µg per mouse. Therefore, the ADCC activities in the sera of mice immunized with the RBD protein were stronger than that of mice vaccinated with the recombinant protein vaccine (Fig. 4e, g). The ADCC response elicited by SARS-CoV-2 RBD- and S2 subunit-immunized mice were compared among VOCs, which revealed a highly comparable level from both RBD (Fig. 4g) and S2 (Fig. 4h) proteins for the four tested strains, although with a slightly higher level obtained from S2 immunization against the B.1.1.7 strain.

## Discussion

While treatment with convalescent plasma derived from recovered individuals is regarded as a potential therapeutic intervention,^21,22^ the medical community is still concerned about the effectiveness of this therapeutic strategy for treating COVID-19, partially due to the lack of understanding of whether convalescent plasma will interfere with anti-SARS-CoV-2 immunity. Here, by analyzing ADCC effect during the COVID-19 clinical course and in relation to the disease severity and clinical outcomes, we found that the peak level of ADCC was around 2 weeks after disease development, which was earlier than the peak time of Nabs that was around 3 weeks after disease development. ADCC activity remained relatively stable from 6 to 12 months, until the last sampled point at 462 days post infection. This finding indicates the application of ADCC as a marker of infection than Nab, which extends the window beyond that inferred from neutralizing activity.

ADCC has been observed to play a critical role in clearing infection in vivo, even in the context of existing potent neutralization activity.^23^ The current study revealed elevated ADCC activity in severe disease than in mild disease, which remained consistent across clinical course and convalescence, raising the possibility that ADCC response may contribute to pathology. An elevated ADCC response has been reported to induce inflammation during viral infection^24^ and whether the ADCC response also correlates to immune-pathogenesis in COVID-19 warrants further investigation. On the other hand, a stronger ADCC response could also be the result of prolonged antigen exposure due to high viral loads in severe patients. However, when severe patients were analyzed separately, those survived from severe disease displayed higher ADCC activity than those deceased, with significance attained at the ADCC peak time, i.e., after day 11 of disease onset. It is possible that timely induction of ADCC might render an improved clinical outcome, but with the advantage limited to those with severe manifestations. A previous study has reported a correlation between delayed production of Nabs and fatal COVID-19.^25^ This might be explained by a similar mechanism by which the timing of antibody production is critical to determine the clinical outcome, i.e., patients who only develop delayed Nab production or those with early waning of ADCC activity might succumb to a more severe infection. Possibly, the cells that mediate ADCC also become functionally compromised in early stages of infection, thereby depriving the host of the potential benefits of this process. Further studies with a larger sample size are warranted to test this hypothesis.

Increasing evidence suggests that some newly emerged SARS-CoV-2 mutants resist neutralization by antibodies elicited by the early pandemic wild-type virus. Recent studies have shown that the neutralizing activity of post-vaccination serum is compromised against new SARS-CoV-2 variants. The K417N+E484K+N501Y triple mutant in the B.1.351 variant significantly reduces the neutralizing activity of convalescent and post-vaccination sera.^26,27^ Here we have shown that highly comparable ADCC was elicited against VOC and the D614G reference strain from infected patients. In the mouse model, immunization with pseudoviruses of SARS-CoV-2 VOCs (which included B.1.17, B.1.351, and P.1 variants) triggered comparable ADCC levels and in a similar level to that of the D614G reference strain. This verified the important role that Fc-dependent ADCC effector functions in SARS-CoV-2 infection in vivo, and particularly in the context of emerging neutralization-resistant viral variants. Still, more in-depth investigation is warranted to explore the difference from the immunization program and sampling time.

Another notable finding was that the ADCC responses to SARS-CoV-2 exhibited variations that depended on the antigen. Importantly, ADCC activities can be mediated by diverse epitope specificities, with a higher level against both the RBD and S2 subunit of the S protein compared to that against NTD, and an even higher level against the S2 subunit, which was in contrast with the Nab and binding IgG antibody of which higher levels were induced against RBD. Similar to our results, Pinto et al. found that non-RBD-binding Ab S306 lacks the ability to mediate neutralizing activity but mediates ADCC.^28^ In SARS-CoV-2-naive individuals, robust ADCC activity but weak neutralizing activity were induced after a single dose of SARS-CoV-2 vaccine BNT162b2, which indicated that the non-Nabs triggered by the COVID-19 vaccine had ADCC activity.^29^ In the present study, antibodies induced by the RBD showed a stronger neutralizing activity with a weaker ADCC activity, whereas those elicited by S2 antigen showed a weaker neutralizing activity and stronger ADCC activity. Collectively, these results suggest that non-Nabs against SARS-CoV-2 have a potential ADCC activity. Future study may identify key non-neutralizing epitopes that induce robust ADCC responses against COVID-19, which will shed light on the humoral immunity triggered by SARS-CoV-2.

It has been reported that asymptomatic individuals possess antibodies against the SARS-CoV-2 S protein.^30^ Here we also observed elicitation of ADCC in asymptomatic individuals. Unfortunately, the asymptomatic individuals were not followed up to elucidate the long-term pattern of ADCC.

The current findings also complement the available knowledge on the presence of Nabs during early convalescence.^31–33^ Previous studies have reported that Nabs in most SARS-CoV-2-infected patients persists for up to 11 months after infection.^34^ Here we observed persistence of detectable Nabs for as long as 1 year, with only a mild decline. Before the current study, there was controversial evidences of a protective or detrimental roles for Nab in SARS-CoV-2 infection in multiple animal models, as well as in COVID-19 patients.^35,36^ Passive transfer of mAbs can protect animal models from SARS-CoV-2 challenge,^37–39^ which suggests that antibody neutralization may provide protection against SARS-CoV-2 infection.^40^ In contrast to the beneficial role of Nabs, we found higher SARS-CoV-2-specific Nabs in severe vs. mild disease, although the sampling was limited to early infection, which is in line with previous studies that have reported a positive correlation between the magnitude of the antibody response and disease severity.^41–44^

Despite the advances discussed above, we acknowledge some limitations in the present study. First, commercial ADCC bioassay effector cells were used to profile ADCC responses to SARS-CoV-2 infection in this study. Although these effector cells have been used to evaluate the ADCC activity,^1,28,45,46^ it should be noted that using innate effector cells may provide a better comprehensive overview of the ADCC activity associated with SARS-CoV-2 infection. Second, because of limited experimental resources, we did not conduct in vivo experiments to verify the contribution of ADCC to SARS-CoV-2 infection.

In conclusion, we have demonstrated induction of ADCC activity by the RBD and S2 subunit of the S protein and identified ADCC as a correlate of the host immune response to SARS-CoV-2 infection or vaccination. FcγR-binding ADCC functions decay at a slow rate after recovery from COVID-19, which may act as an effective non-Nab response elicited by infection or vaccination. This finding might help to improve our understanding of the role of ADCC in disease development, and may provide new insights into the underlying mechanisms of antibody-mediated immunity. This knowledge may also improve the development of vaccine and therapeutic strategies to provide highly effective humoral defense and to achieve optimal efficacy and safety of vaccines and therapeutics for COVID-19.

## Materials and methods

### Study cohorts and serum samples

The cohort study was performed on patients with polymerase chain reaction (PCR)-confirmed SARS-CoV-2 infection that had been treated in three designated hospitals for COVID-19 in Hubei province, which included Wuhan Jinyintan Hospital and Tongji Hospital of Huazhong University of Science and Technology in Wuhan city and Huangmei People’s Hospital in Huanggang city. The cohort included 234 patients and 21 asymptomatic individuals with SARS-CoV-2 infection. Their median age was 49 years (IQR: 33–65), and 164 (64%) were male. These COVID-19 patients had been diagnosed and admitted in accordance with the Guideline of the National Health Commission of China, i.e., positive results for SARS-CoV-2 RNA by performing real-time reverse transcriptase PCR (RT-PCR) on the nasopharyngeal swab and aspirate as guided.^47^ The case cohort was established during hospitalization and followed up after discharge from hospitals, on the basis of which the patients were prospectively sampled at around 1, 3, 6, and 12 months after infection. At each visit, blood samples were collected to obtain serum, which were stored at −80 °C until tested in a batch. In total, 139 samples were collected in the acute phase (≤14 days after onset of symptoms) and 202 (59.24%) samples were collected in the convalescent phase (>14 days from onset of symptoms, range 15–462 days). Twenty-seven patients provided ≥3 specimens at >30 days apart, (67% male, median 55, IQR, 40–62 years old, 12 with severe disease, and 15 with moderate disease). Samples were collected from asymptomatic individuals at 2–15 days after the first positive test for SARS-CoV-2 RNA. It is important to note that, as with symptomatic infection, the definition of the clinical type (mild, moderate, and severe infection) was revised during the study periods. Therefore, all clinical records of the patients during their entire hospitalization were re-checked and re-defined for the clinical phenotype classification according to the most updated diagnostic and treatment guidelines for SARS-CoV-2 issued by the Chinese National Health Committee (Version 8).^48^ Briefly, mild infection was defined as an individual who had mild clinical symptoms without radiological signs of pneumonia. Moderate infection was defined as meeting the following criteria: (i) fever and respiratory symptoms; (ii) radiological signs of pneumonia. Severe infection was defined as satisfying at least one of the following items: (i) breathing rate ≥30/min; (ii) pulse oximeter oxygen saturation (SpO_2_) ≤93% at rest; (iii) ratio of partial pressure of arterial oxygen (PaO_2_) to the fraction of inspired oxygen (FiO_2_) ≤300 mm/Hg (1 mm/Hg = 0.133 kPa). Asymptomatic infection was determined as a positive result for SARS-CoV-2 RNA by RT-PCR without showing any relevant symptoms. Patients were also stratified by clinical outcomes into survived and fatal, which were retrieved from medical records and verified by performing a follow-up visit of patients who had discontinued therapy or had been discharged from hospital because of adverse clinical progression. The research protocol was approved by the human ethics committee of the hospitals, and all participants or their guardians had provided written informed consent.

### Cell lines

Huh-7 (human, liver) and 293T (human, kidney) cell lines were obtained from the Japan Research Biological Resources Collection and American Type Culture Collection, respectively. 293T-spike cells were 293T cells (Sino Biological, China) that stably expressed SARS-CoV-2 S protein (WuHan wild-type strain, GenBank: MN908947). These cells were cultured in Dulbecco’s modified Eagle medium supplemented with 10% fetal bovine serum (FBS), 2 Mm L-glutamine, 100 U/ml penicillin-streptomycin, and 20 mM *N*-2-hydroxyethylpiperazine-*N*-2-ethane sulfonic acid. ADCC bioassay effector cells (Jurkat-FcγRIIIa-NFAT-Luc) and murine FcγRIII ADCC effector cells were obtained from Promega (USA) and cultured in RPMI 1640 medium (Gibco, USA) with 10% FBS, 100 μg/ml hygromycin, 250 μg/ml Antibiotic G-418 Sulfate Solution, 1 mM sodium pyruvate, and 0.1 mM MEM nonessential amino acids. All cell lines were cultured at 37 °C with 5% CO_2_ and passaged every 2–3 days.

### Sera from immunized mice

The animal experimental protocol was approved by the Ethical Review Committee for Animal Welfare of the National Institutes for Food and Drug Control. SARS-CoV-2 NTD-, RBD-, and S2 subunit protein-immunized mouse sera were obtained by immunizing specific pathogen-free BALB/c mice with purified proteins (ACROBiosystems, China). The corresponding protein (20 μg) was mixed with an equal amount of aluminum adjuvant. For each protein, ten mice were subcutaneously immunized every other week. Immunized mouse sera were collected from immunized mice at 28 days following the third immunization. Pre-immune serum was used as negative control.

Pseudotyped virus-immunized mouse sera were obtained by immunization with purified SARS-CoV-2 plasmid and pseudotyped viruses of various strains. On day 0, 10 mice in each group were intramuscularly immunized with purified SARS-CoV-2 plasmid comprising D614G reference strain, Alpha (B.1.1.7) variant, Beta (B.1.351) variant, or Gamma (P.1) variant and an aluminum adjuvant (50 µg per mouse). Pseudotyped viruses of the corresponding SARS-CoV-2 variants were intraperitoneally injected into mice on days 14 and 28 (6 × 10^5^ TCID_50_ per mouse). Blood samples were collected at 28 days after the third immunization.

Serum samples collected from mice vaccinated with inactivated vaccine (*n* = 20), adenovirus vector vaccine (*n* = 20), mRNA vaccine (*n* = 20), or recombinant protein subunit vaccine (*n* = 20) were kindly provided by Kexing Biopharm, CanSino Biologics Inc., Stemirna Therapeutics, and Anhui Zhifei Longcom Biopharmaceutical Co., Ltd., respectively.

### ADCC measurements

Commercial ADCC bioassay effector cells, Jurkat-FcγRIII-NFAT-Luc reporter cells, were used to evaluate ADCC activity.^1,28,45,46^ This ADCC assay measures the ability of serum to activate the NFAT (nuclear factor of activated T cells) pathway through FcγRIII (the pathway that initiates ADCC in NK cells) in the presence of target antigens expressed on the 293T cell surface. The ADCC reporter assay was performed in accordance with the manufacturer’s instructions (Promega). ADCC assays of sera from COVID-19 patients and sera from pseudovirus of SARS-CoV-2 VOCs and NTD-, RBD-, and S2 protein-immunized mice were performed using 293T-spike cells that stably expressed SARS-CoV-2 S protein as targets. ADCC assays of sera from mice vaccinated with different vaccines were performed using the 293T cell transiently transfected with SARS-CoV-2 S protein as targets.

To obtain SARS-CoV-2 S protein-overexpressing cells, 293T cells were transfected with SARS-CoV-2-spike_pcDNA-3.1 comprising D614G reference strain, B.1.1.7 variant, B.1.351 variant, or P.1 variant using Lipofectamine 3000 (Invitrogen, USA). After 24 h of culture at 37 °C with 5% CO_2_, the cells were collected for immunostaining with SARS-CoV-2 mAb. An Alexa-488-labeled goat anti-human IgG (H+L) secondary antibody (Invitrogen) was used for detection. The fluorescent signal was examined using flow cytometer (BD FACS CantoTM II, BD Biosciences, USA).

For all ADCC assays, target cells (2.5 × 10^4^ per well) were incubated with serial dilutions of serum samples at 37 °C for 30 min. ADCC effector cells (7.5 × 10^4^ per well) were then added to each well. After incubation at 37 °C for 12 h, the relative light unit (RLU) was detected in accordance with the instruction manual provided by PerkinElmer (Waltham, MA). Fold of induction was calculated as follow: RLU (induced − background)/RLU (no serum control − background).

### Pseudovirus-based neutralization assay

The pseudotyped virus neutralization assay was measured by the reduction in Luc gene expression and performed in accordance with the method described in our previous studies.^49,50^ In brief, serially diluted samples were incubated with pseudotyped virus (6 dilutions in a 3-fold step-wise manner) in duplicate for 1 h at 37 °C together with the virus control and cell control wells in sextuplicate. Thereafter, 2 × 10^4^ freshly trypsinized Huh-7 cells were added to each well of the 96-well plate. After 24 h of incubation at 37 °C with 5% CO_2_, the RLU was measured in accordance with the instruction manual provided by PerkinElmer (Waltham, MA). The ED_50_ (median effective dilution) was calculated using the Reed–Muench method. Cutoff values were set as 30 and 50 for human and mouse serum samples, respectively.^49^

### Enzyme-linked immunosorbent assay (ELISA)

To determine the binding avidity of mouse serum samples, serially diluted mouse serum samples were incubated at 37 °C in a 96-well ELISA microplate (Thermo Fisher, USA) coated with 0.1 µg/well SARS-CoV-2 S protein. After 1 h of incubation, the plates were washed three times with wash buffer and then incubated with a 1:4000 dilution of horseradish peroxidase-conjugated goat anti-human IgG (Genscript, China) for 1 h at 37 °C. After five washes, 100 µl of freshly prepared 3,3’,5,5’-tetramethylbenzidine substrate (Wantai, China) was added to each well, followed by incubation for 15 min at room temperature in the dark, and then 50 μl/well of 2 M H_2_SO_4_ was added to stop the reaction. The result is presented as absorbance read at 450 nm subtracted from absorbance at 620 nm.

### Statistical analysis

Descriptive statistics were calculated for all variables. A continuous variable was summarized as the mean and standard deviation or the median and IQR, and a categorical variable was summarized as the frequency and proportion. To determine differences between two groups, *χ*^2^ test, Fisher’s exact test, or nonparametric test was used where appropriate. One-way analysis of variance was used to compare differences among three or more groups. Generalized estimating equation (GEE) was performed to identify variables that were associated with the levels of ADCC fold induction and neutralization ED_50_. GEE was also used to explore associations between the levels of ADCC fold induction and neutralization ED_50_ and the risk of fatal outcome. The variables of age, sex, and days from symptom onset were adjusted. All statistical analyses were performed using the Stata 17.0 (Stata Corp LP, College Station, TX, USA) and R 3.5.3 (R Development Core Team, Vienna, Austria) softwares. Two-sided *P* < 0.05 was considered statistically significant.

## Disclaimer

This study has neither been presented nor submitted or accepted anywhere.

## Supplementary information

Supplementary Materials

## Data Availability

The datasets in this study are available from the corresponding author upon reasonable request.
