# Supplementary material for: Antibody-dependent cellular cytotoxicity response to SARS-CoV-2 in COVID-19 patients
Source: Signal Transduct Target Ther. 2021 Sep 24;6:346. doi: 10.1038/s41392-021-00759-1 (PMC8463587; doi:10.1038/s41392-021-00759-1)
Supplement: Supplementary file 1 — Supplementary Materials [file 41392_2021_759_MOESM1_ESM.docx]

Supplementary Materials for

Antibody-dependent cellular cytotoxicity response to SARS-CoV-2 in COVID-19 patients

Yuanling Yu^#,1^, Meiyu Wang^#,1,2^, Xiaoai Zhang^#,3^, Shufen Li^#,4^, Qingbin Lu^5^, Haolong Zeng^6^, Hongyan Hou^6^, Hao Li^3^, Mengyi Zhang^1^, Fei Jiang^1^, Jiajing Wu^1^, Ruxia Ding^1^, Zehua Zhou^1^, Min Liu^7^, Weixue Si^8^, Tao Zhu^8^, Hangwen Li^9^, Jie Ma^9^, Yuanyuan Gu^9^, Guangbiao She^10^, Xiaokun Li^3^, Yulan Zhang^4^, Ke Peng^4,11^, Weijin Huang*^,1^, Wei Liu*^,3^, Youchun Wang*^,1,2^.

Correspondence to: huangweijin@nifdc.org.cn, lwbime@163.com, wangyc@nifdc.org.cn

**This file includes:**

Figures. S1 to S2


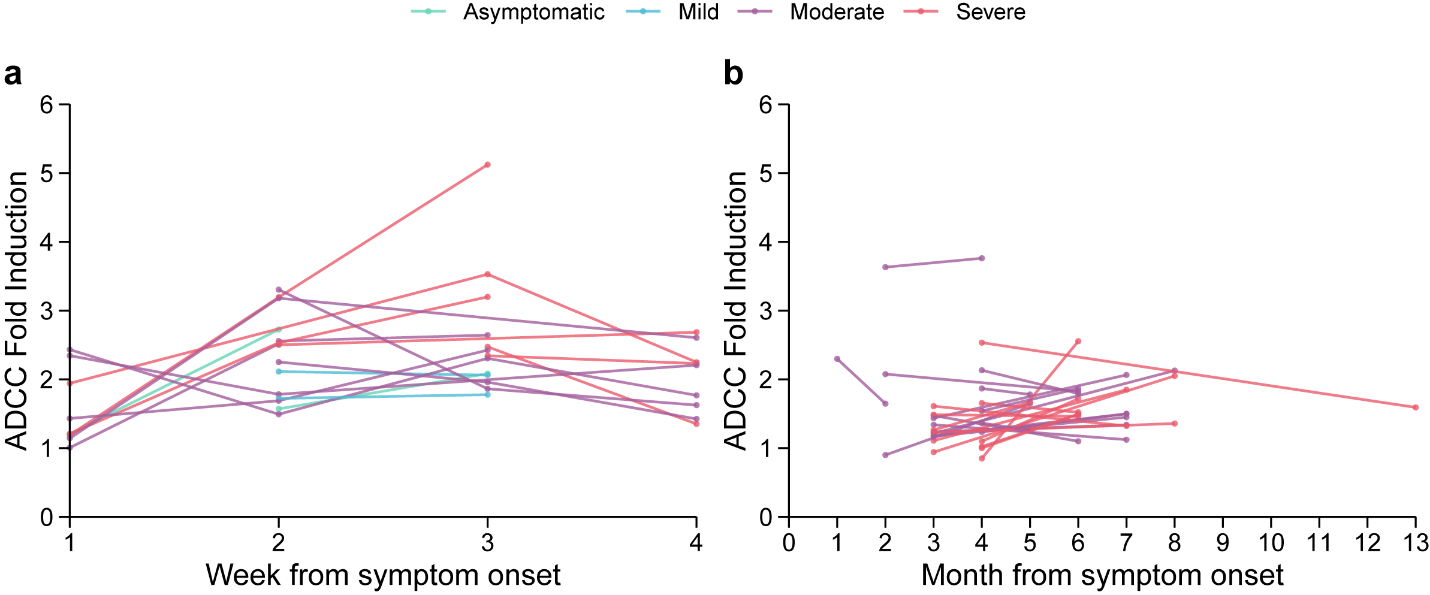


Figure. S1. Dynamics of ADCC activity in COVID-19 patients.

(a) Kinetics of ADCC fold induction in individual COVID-19 patients at 1 month after symptom onset. (b) Kinetics of ADCC activity in individual COVID-19 patients at 1 year after symptom onset. Each curve represents one patient. Green curves indicate asymptomatic COVID-19 patients; blue curves indicate mild COVID-19 patients; purple curves indicate moderate COVID-19 patients; red curves indicate severe COVID-19 patients. ADCC, antibody-dependent cell-mediated cytotoxicity. COVID-19, coronavirus disease 2019.


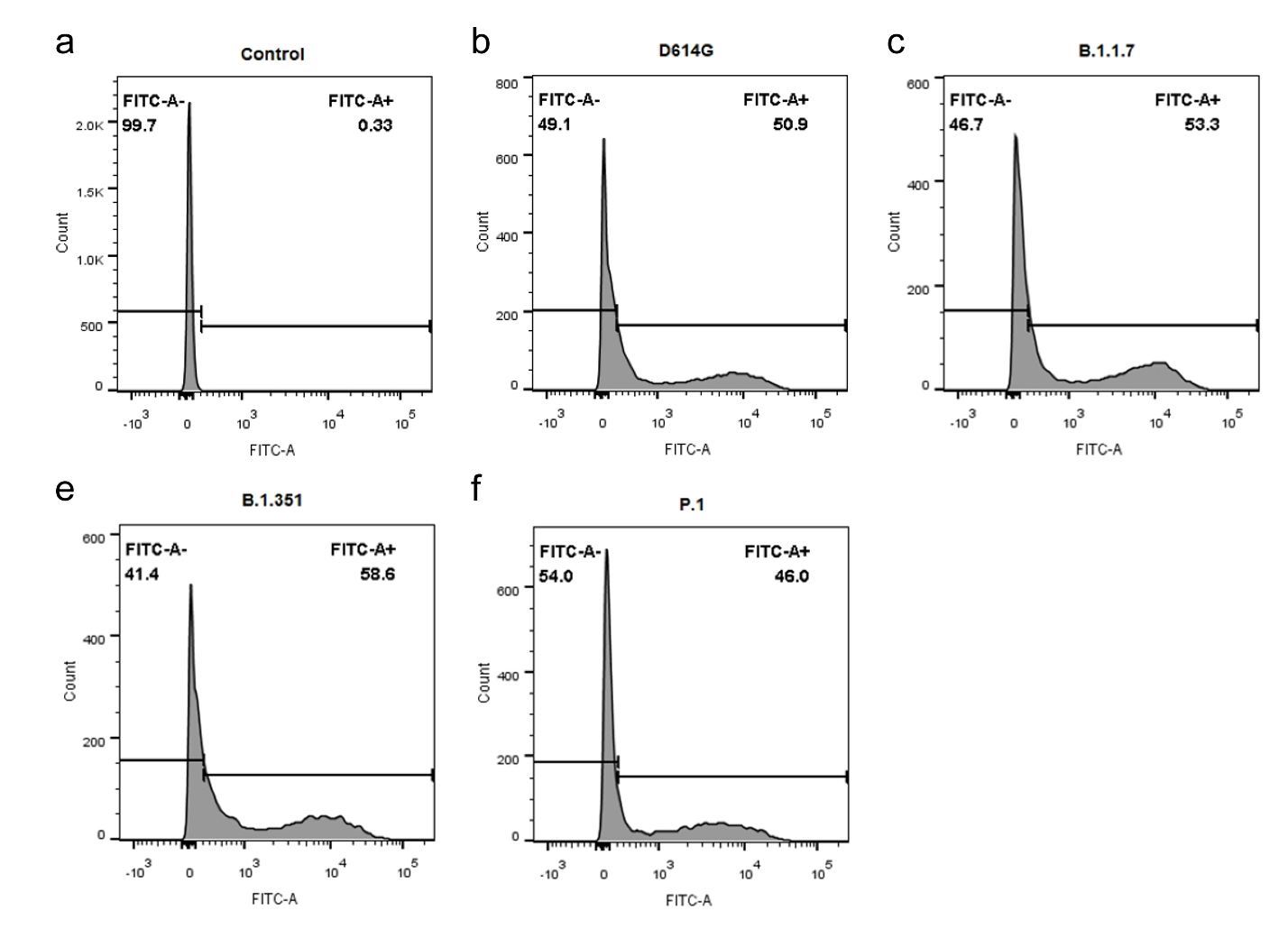


Figure. S2. Expression levels of spike proteins of different variants on surfaces of transfected 293T cells.

Cell surface expression levels of spike proteins were assessed by flow cytometry. The FITC-A+ value in upper right corner represents the proportion of spike-expressing cells.
